# Oral Prevalence of *Akkermansia muciniphila* Differs among Pediatric and Adult Orthodontic and Non-Orthodontic Patients

**DOI:** 10.3390/microorganisms11010112

**Published:** 2023-01-01

**Authors:** Ching Shen, James Brigham Clawson, Justin Simpson, Karl Kingsley

**Affiliations:** 1 Department of Advanced Education in Orthodontic Dentistry, School of Dental Medicine, University of Nevada—Las Vegas, 1700 W. Charleston Boulevard, Las Vegas, NV 89106, USA; 2 Department of Clinical Sciences, School of Dental Medicine, University of Nevada—Las Vegas, 1700 W. Charleston Boulevard, Las Vegas, NV 89106, USA; 3 Department of Biomedical Sciences, School of Dental Medicine, University of Nevada—Las Vegas, 1001 Shadow Lane, Las Vegas, NV 89106, USA

**Keywords:** *Akkermansia muciniphila*, saliva screening, orthodontic, oral prevalence

## Abstract

*Akkermansia muciniphila* (AM) is one of many highly abundant intestinal microbes that influences homeostasis and metabolic disorders and may also play a role in oral disorders. However, there is little evidence regarding the oral prevalence of this organism. Based upon this lack of evidence, the primary goal of this project is to survey an existing saliva repository to determine the overall prevalence of this organism and any associations with demographic or patient characteristics (age, sex, body mass index, race/ethnicity, orthodontic therapy). Using an approved protocol,, a total *n* = 141 pediatric samples from an existing saliva repository were screened using qPCR revealing 29.8% harbored AM with nearly equal distribution among males and females, *p* = 0.8347. Significantly higher percentages of pediatric, non-orthodontic patients were positive for AM (42.3%) compared with age-matched orthodontic patients (14.3%)—which were equally distributed among non-orthodontic males (42.1%) and non-orthodontic females (42.5%). In addition, analysis of the adult samples revealed that nearly equal percentages of males (18.2%) and females (16.7%) harbored detectable levels of salivary AM, *p* = 0.2035. However, a higher proportion of non-orthodontic adult samples harbored AM (21.3%) compared to orthodontic samples (12.8%, *p* = 0.0001), which was equally distributed among males and females. These results suggest that both age and the presence of orthodontic brackets may influence microbial composition and, more specifically, are associated with reduction in AM among both pediatric and adult populations from their baseline levels.

## 1. Introduction

*Akkermansia muciniphila* is one of many highly abundant human intestinal microbes that may have the ability to significantly influence homeostasis and metabolic disorders [1,2]. For example, some evidence has demonstrated that decreased abundance of the mucin-degrading *Akkermansia* allows for dysbiosis among other microbial competitors that negatively affect host metabolic syndrome and immune system responses [3,4]. Systematic reviews of this evidence have confirmed that reductions in *Akkermansia* are highly associated with metabolic syndrome as the inflammation-reducing properties of this microbe are lost, associations that may also be related to other disorders including obesity, hyperlipidemia and hypertension [5,6].

The functional ability of this microorganism to modulate inflammation, basal metabolism and other metabolic functions has led to many studies evaluating the potential for its use as a therapeutic treatment option [7,8]. Growing evidence has demonstrated the benefits and impacts of *Akkermansia* used as a probiotic to reduce inflammation and restore microbial homeostasis and equilibrium in clinical settings [9,10,11]. However, recent studies have demonstrated that changes to the oral microbiota, most notably with *Akkermansia*, not only alter oral health but also precede and subsequently contribute to the development or prevention of these metabolic disorders [12,13].

More specifically, the presence of *Akkermansia* appears to down-regulate the production of inflammatory cytokines such as IL-10 and IL-12, as well as other inflammatory biomarkers associated with *Porphyromonas gingivalis*-associated periodontitis [14,15]. Additionally, other studies have now revealed that administration of *Akkermansia* may reduce both periodontal and systemic inflammation in a dose-dependent manner [16,17]. However, the only studies of prevalence to date have focused on evaluation of microbial composition or changes within the gut and intestinal tracts without any corresponding studies of oral microbial prevalence [18,19,20].

Given the ability of these microorganisms to influence oral as well as systemic disorders, it is surprising how little evidence is available regarding the prevalence and oral ecology of this organism [21]. Studies from this group have created a saliva biorepository that has been successfully used to conduct molecular screenings for other novel oral pathogens, including the cariogenic bacterium *Scardovia wiggsiae* and periodontal pathogen *Selenomonas noxia* [22,23,24]. Based upon this information, the primary goal of this project was to survey this existing saliva biorepository to determine the overall prevalence of *Akkermansia muciniphila* and any associations with demographic or patient characteristics, such as age, sex, race or ethnicity, and orthodontic treatment status.

## 2. Materials and Methods

### 2.1. Human Subjects Approval

This was a retrospective study involving the use of an existing biorepository. The protocol for this study was submitted, reviewed and approved by the Institutional Review Board (IRB) and the Office for the Protection of Research Subjects (OPRS) at the University of Nevada, Las Vegas (UNLV) as Research Exempt under Protocol #1717625-1 “Retrospective analysis of microbial prevalence from DNA isolated from saliva samples originally obtained from the University of Nevada, Las Vegas (UNLV) School of Dental Medicine (SDM) pediatric and clinical population” on 3 March 2021.

### 2.2. Original Collection Protocol

The study protocol used for the original collection of clinical saliva samples was reviewed and approved under protocol OPRS#1305-4466M “The Prevalence of Oral Microbes in Saliva from the UNLV School of Dental Medicine Pediatric and Adult Clinical Population” in 2013. In brief, adult and pediatric patients who agreed to participate provided Informed Consent and Pediatric Assent, if applicable. Up to 5.0 mL was collected in sterile polypropylene tubes, which were labeled with non-duplicated, randomly generated numbers to avoid the collection of any personal or patient-specific information.

Inclusion criteria were patients of record at UNLV-SDM who agreed to participate and provide Informed Consent and/or Pediatric Assent. Exclusion criteria included any person not a patient of record at UNLV-SDM and any patient who declined to participate. Samples were stored at −80 °C in a secured biomedical laboratory freezer. Basic demographic information was concurrently collected including patient age, sex, race or ethnicity, as well as orthodontic treatment status.

### 2.3. DNA Isolation and Analysis

DNA was isolated from all clinical samples using the phenol:chloroform extraction method using TRIzol DNA isolation reagent from ThermoFisher Scientific (Fair Lawn, NJ, USA), as previously described [22,23,24]. Briefly, samples were thawed and 400 uL of saliva was removed and placed into a sterile microcentrifuge tube with an equal volume of TRIzol reagent and triturated before adding 200 uL of chloroform and incubated for ten minutes on ice. Samples were then centrifuged at 12,000× *g* or relative centrifugal force (RCF) for 15 min using an Eppendorf Refrigerated Microcentrifuge obtained from Fisher Scientific (Fair Lawn, NJ, USA). The upper aqueous phase was transferred to a sterile microcentrifuge tube with an equal volume of isopropanol to precipitate the DNA and mixed thoroughly. Samples were centrifuged for 10 min and isopropanol was removed. Samples were washed with molecular grade Ethanol and centrifuged for five minutes. Ethanol was removed and samples were resuspended using nuclease-free distilled water.

DNA was screened using the NanoDrop 2000 Spectrophotometer from Fisher Scientific (Fair Lawn, NJ, USA). Absorbance readings at A260 nm and A280 nm were used to determine the purity and concentration of DNA. Samples with sufficient quantity (>10 ng) and quality as determined by the A260:A280 ratio (>1.65) were selected for qPCR screening.

### 2.4. qPCR Screening

Screening of samples was performed using the QuantStudio Real-Time Polymerase Chain Reaction (PCR) system from Applied Biosciences (Waltham, MA, USA). Screening qPCR reactions utilized SYBR Green qPCR Master Mix from ThermoFisher Scientific (Fair Lawn, NJ, USA), which consisted of ABsolute SYBR Green (12.5 uL), nuclease-free water (7.5 uL), forward and reverse primers (1.75 uL each), and sample DNA (1.5 uL) diluted to 1.0 ng/uL for a total reaction volume of 25 uL. Cycle specifications included activation of the enzyme at 95 °C for 15 min followed by 40 cycles of denaturation at 95 °C (15 s), annealing using each primer pair-specific temperature (30 s), with final extension at 72 °C (30 s). Validated primer sets included [22,23,24,25,26,27,28,29]:

Positive control, bacterial 16S rRNA

Forward 16S rRNA primer: 5′-ACG CGT CGA CAG AGT TTG ATC CTG GCT-3′; 27 nt, 56% GC content, Tm = 76 °C

Reverse 16S rRNA primer: 5′-GGG ACT ACC AGG GTA TCT AAT-3′; 21 nt, 48% GC content, Tm = 62 °C

*Akkermansia muciniphila* (AM)

Forward AM primer: 5′-CAG CAC GTG AAG GTG GGG-3′; 18 nt, 67% GC content, Tm = 69 °C

Reverse AM primer: 5′-CCT TGG GGT TGG CTT CAG AT-3′, 20 nt, 55% GC content, Tm = 68 °C

*Aggregatibacter actinomycetemcomitans* (AA)

Forward AA primer, 5′-ATT GGG GTT TAG CCC TGG T-3′; 19 nt, 53% GC, Tm = 67 °C

Reverse AA primer, 5′-GGC ACA AAC CCA TCT CTG A-3′; 19 nt, 53%GC, Tm = 65 °C

*Fusobacterium nucleatum* (FN)

Forward FN primer; 5′-CGC AGA AGG TGA AAG TCC TGT AT-3′; 23 nt, 48% GC, Tm = 67 °C

Reverse FN primer; 5′-TGG TCC TCA CTG ATT CAC ACA GA-3′; 23 nt, 48% GC, Tm = 68 °C

*Selenomonas noxia* (SN)

Forward SN primer: 5′-TCT GGG CTA CAC ACG TAC TAC AAT G-3′; 25 nt, 48% GC, Tm = 68 °C

Reverse SN primer: 5′-GCC TGC AAT CCG AAC TGA GA-3′; 20 nt, 55% GC, Tm = 68 °C

*Porphyromonas gingivalis* (PG)

Forward PG primer: 5′-TAC CCA TCG TCG CCT TGG T = 3′; 19 nt, 58% GC, Tm = 69 °C

Reverse PG primer: 5′-CGG ACT AAA ACC GCA TAC ACT TG-3′; 23 nt, 48% GC, Tm = 66 °C

### 2.5. Statistical Analysis

Descriptive statistics for demographic variables, including number and percentage of males and females, average age with range, and race or ethnicity were compiled in Microsoft Excel (Redmond, WA, USA). Samples were screened and further categorized as AM-positive and AM-negative for analysis using Chi Square statistics, which is appropriate for non-parametric (categorical data) analysis. A significance level of alpha = 0.05 was used for all calculations.

## 3. Results

A total of *n* = 227 clinical saliva samples were identified for inclusion in this study. Analysis of the samples from the pediatric population (*n* = 141) revealed approximately half were derived from females (51.1%), which closely matched the overall clinic demographics (52.8%), *p* = 0.6891 (Table 1). Evaluation of the racial and ethnic demographic data revealed the majority of samples were derived from minority (non-White) patients (70.2%), which also approximates the overall pediatric clinic population (75.3%), *p* = 0.2482. The average age of the pediatric study sample was 13.14 years, which was significantly higher than the overall clinical population age of 10.44 years mainly due to the original sampling protocol which restricted sample collection to patients age seven and older, *p* = 0.021.

Analysis of the samples from the adult population (*n* = 86) revealed approximately half were derived from females (48.8%), which closely matched the overall clinic demographics (49.1%), *p* = 0.9883 (Table 2). Evaluation of the racial and ethnic demographic data revealed the majority of samples were derived from minority (non-White) patients (62.8%), which also approximates the overall adult clinic population (65.4%), *p* = 0.0893. Finally, the average age of the adult study sample was 46.35 years, which was significantly higher than the overall clinical population age of 42.31 years, *p* = 0.028.

The DNA isolated from the pediatric and adult saliva samples was screened using spectrophotometric analysis to determine the suitability of each sample for qPCR screening (Table 3). These data revealed that the average DNA concentration from the pediatric saliva samples was sufficient for qPCR screening (average 481.2 ng/uL), which was within the range specified by the manufacturer for isolation from biological samples (100–1000 ng/uL). In addition, the quality of samples was also suitable for qPCR screening (A260:A280 ratio = 1.73 average.) Similarly, the concentration (434.1 ng/uL) and quality (A260:A280 ratio = 1.75 average) of DNA from adult saliva samples was also sufficient for qPCR screening and analysis.

**Figure 1:**
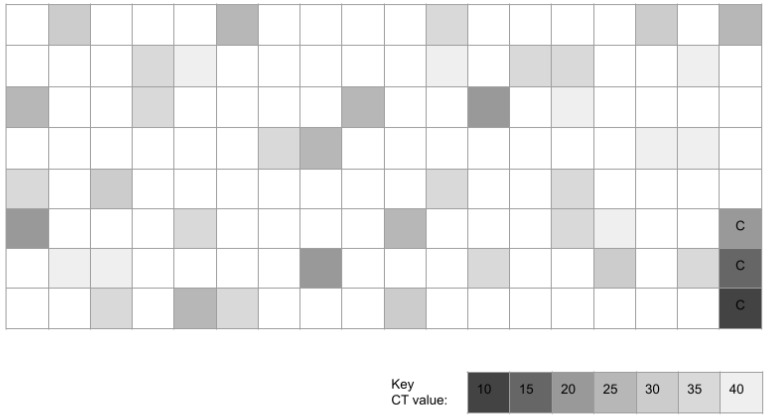
Heat map of qPCR pediatric study sample screening for *Akkermansia muciniphila* (AM). This screening revealed *n* = 42/141 or 29.8% of samples harbored DNA specific for AM. The majority of these samples (*n* = 28/42 or 66.7%) exhibited AM-specific DNA levels corresponding to low (C36–C40) or moderately low (C31–C35) cycle threshold detection values. C = positive control standard curve data for 16S rRNA.

Molecular screening of pediatric study samples revealed *n* = 42/141 or 29.8% harbored DNA specific for *Akkermansia* or AM (Figure 1). Approximately one-third exhibited cycle threshold detection levels in the high (C16–C20, *n* = 3/42 or 7.1%), moderate-high C21–C25, *n* = 7/42 or 16.7%) or moderate (C26–C30, *n* = 4/42 or 9.5%) range. In contrast, two-thirds (*n* = 28/42 or 66.7%) exhibited cycle threshold detection levels in the low-moderate (C31–C35, *n* = 15/42 or 35.7%) or low (C36–C40, *n* = 13/42 or 31.0%) range.

More detailed analysis of these pediatric samples revealed that nearly equal percentages of males (*n* = 21/69 or 30.4%) and females (*n* = 21/72 or 29.2%) harbored detectable levels of salivary AM, *p* = 0.8347 (Table 4). Interestingly, sorting by orthodontic status revealed that a higher proportion of non-orthodontic samples harbored AM (*n* = 33/78 or 42.3%) than orthodontic samples (*n* = 9/63 or 14.3%). More specifically, more males (*n* = 16/38 or 42.1%) and females (*n* = 17/40 or 42.5%) from non-orthodontic samples harbored AM than age-matched orthodontic samples from males (*n* = 5/31 or 16.1%) or females (*n* = 4/32 or 12.5%), *p* = 0.0001.

**Figure 2:**
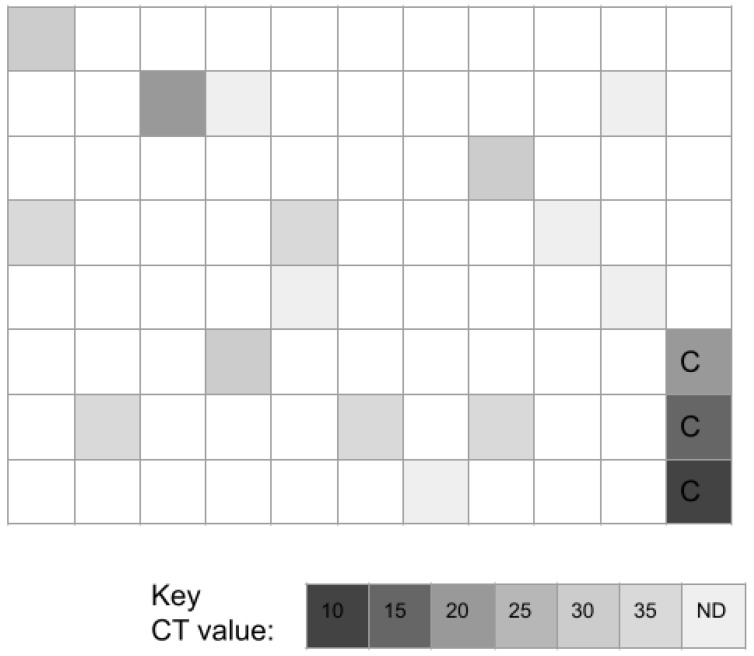
Heat map of qPCR adult study sample screening for *Akkermansia muciniphila* (AM). This screening revealed *n* = 15/86 or 17.4% of samples harbored DNA specific for AM. The majority of these samples (*n* = 11/15 or 73.3%) exhibited AM-specific DNA levels corresponding to low (C36–C40) or moderately low (C31–C35) cycle threshold detection values. C = positive control standard curve data for 16S rRNA.

Molecular screening of adult study samples revealed *n* = 15/86 or 17.4% harbored DNA specific for AM (Figure 2). Only four samples exhibited cycle threshold detection levels in the high (C16–C20, *n* = 1/15 or 6.7%), moderate-high C21–C25, *n* = 0/15 or 0.0%) or moderate (C26–C30, *n* = 3/15 or 20.0%) range. In contrast, nearly three-fourths (*n* = 11/15 or 73.3%) exhibited cycle threshold detection levels in the low-moderate (C31–C35, *n* = 5/15 or 33.3%) or low (C36–C40, *n* = 6/15 or 40.0%) range.

More detailed analysis of the adult samples revealed that nearly equal percentages of males (*n* = 8/44 or 18.2%) and females (*n* = 7/42 or 16.7%) harbored detectable levels of salivary AM, *p* = 0.2035 (Table 5). Analysis of these data using orthodontic status revealed that a higher proportion of non-orthodontic, adult samples harbored AM (*n* = 10/47 or 21.3%) than orthodontic samples (*n* = 5/39 or 12.8%). More specifically, more adult males (*n* = 5/24 or 20.8%) and females (*n* = 5/23 or 21.7%) from non-orthodontic samples harbored AM than age-matched orthodontic samples from males (*n* = 3/20 or 15%) or females (*n* = 2/19 or 10.5%), *p* = 0.0001.

**Figure 3:**
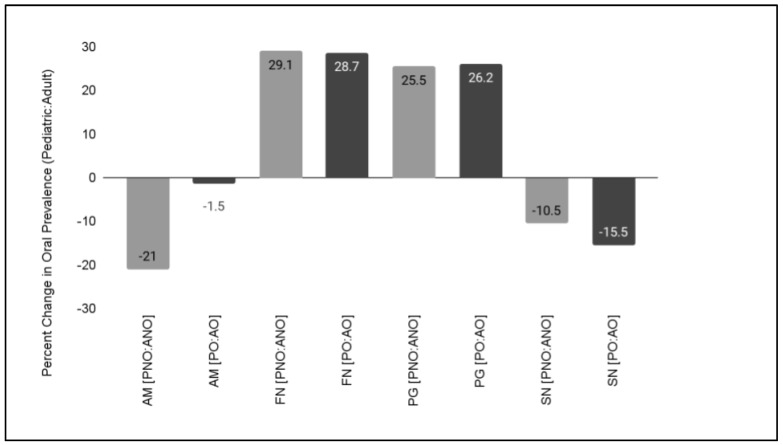
Age-associated difference in oral microbial prevalence. Decreased oral prevalence between pediatric and adult samples was observed for *Akkermansia muciniphila* (AM) with the largest difference observed between the non-orthodontic samples. In addition, other age-related changes were observed with *Fusobacterium nucleatum* (FN), *Porphyromonas gingivalis* (PG) and *Selenomonas noxia* (SN), although those differences were more similar in direction (positive or negative) and magnitude between orthodontic and non-orthodontic samples.

To determine if the changes in oral prevalence were affected by age, differences between the pediatric and adult samples were analyzed (Figure 3). These data demonstrated that a large difference in the oral prevalence of AM was observed between the pediatric, non-orthodontic (42.3%) and adult, non-orthodontic (21.3%) samples at −21%. Further analysis of prevalence among pediatric orthodontic (14.3%) and adult orthodontic (12.8%) revealed a slight difference of only −1.5%.

To determine if these age-associated effects were restricted to this oral microbial population, previously collected data from other studies of Gram-negative oral microbes *Fusobacterium nucleatum* (FN), *Porphyromonas gingivalis* (PG), and *Selenomonas noxia* (SN) were also plotted and graphed. These data also demonstrated differences between the pediatric and adult samples, such as the differences in FN between the pediatric, non-orthodontic (33%) and adult, orthodontic (62.1%) samples at 29.1% that closely matched the differences between pediatric, orthodontic (38.1%) and adult, orthodontic (66.8%) samples at 28.7%. In addition, differences were observed in the oral prevalence of PG between the pediatric, non-orthodontic (28.9%) and adult, non-orthodontic (54.4%) samples by 25.5% that also closely matched the differences between pediatric orthodontic (45.2%) and adult orthodontic (71.4%) samples at 26.2%. Finally, age-associated differences were observed with the oral prevalence of SN between pediatric non-orthodontic (16.6%) and adult orthodontic (5.5%) samples at −10.5, while differences between pediatric, orthodontic (28%) and adult, orthodontic (12.5%) samples were approximately −15.5%.

**Figure 4:**
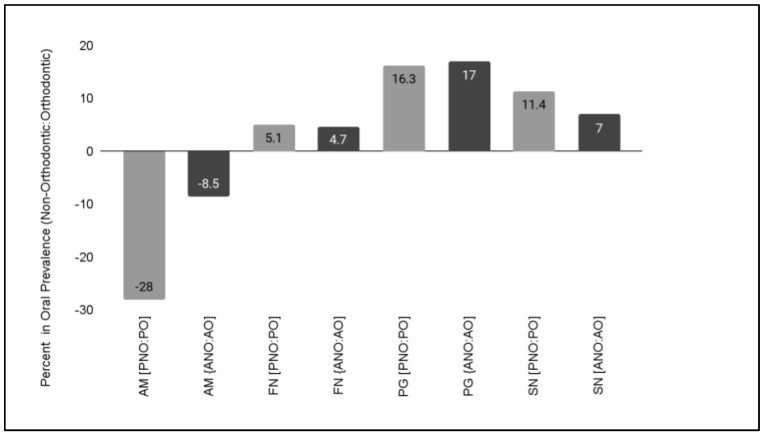
Orthodontic-associated differences in oral microbial prevalence. Decreased oral prevalence between non-orthodontic and orthodontic samples was observed for *Akkermansia muciniphila* (AM) with the largest differences observed between the pediatric samples. In addition, other orthodontic-related changes were observed with *Fusobacterium nucleatum* (FN), *Porphyromonas gingivalis* (PG) and *Selenomonas noxia* (SN), although those differences were more similar in direction (positive or negative) and magnitude between adult and pediatric samples.

To determine if the changes in oral prevalence were correlated with orthodontic therapy, differences between the orthodontic and non-orthodontic samples were analyzed (Figure 4). These data demonstrated that a large difference in the oral prevalence of AM was observed between the pediatric non-orthodontic (42.3%) and pediatric orthodontic (14.3%) samples of −28.0%. In addition, the difference in oral prevalence between adult, non-orthodontic (21.3%) and adult, orthodontic (12.8%) samples was found to be −8.5%.

To determine if these orthodontic-associated differences were restricted to this oral microbial population, data from other studies of Gram-negative FN, PG, and SN were also plotted and graphed. These data also demonstrated differences between the orthodontic and non-orthodontic samples, such as the differences in FN between the pediatric, non-orthodontic (33%) and pediatric, orthodontic (38.1%) samples at 5.1% and between the adult, non-orthodontic (62.1%) and adult, orthodontic (66.8%) samples at 4.7%. In addition, differences were observed in the oral prevalence of PG between the pediatric, non-orthodontic (28.9%) and pediatric, orthodontic (45.2%) samples by 16.3%, as well as the adult, non-orthodontic (54.4%) and adult, orthodontic (71.4%) samples at 17%. Finally, orthodontic-associated differences were also observed with the oral prevalence of SN between pediatric, non-orthodontic (16.6%) and pediatric, orthodontic (28%) samples at 11.4%, while differences between adult, orthodontic (5.5%) and adult, orthodontic (12.5%) samples were approximately 7%.

## 4. Discussion

The principal objective of this study was to evaluate the oral prevalence of *Akkermansia* and to uncover any correlations with patient demographics, such as age, sex, or orthodontic treatment status. This study successfully evaluated more than 225 clinical saliva samples, making it one of the largest and most comprehensive oral prevalence surveys ever undertaken at this institution [24,30]. In addition, due to the lack of evidence regarding oral prevalence of this organism, any studies that provide insight into the factors that may influence the distribution within populations could help us to understand how and when this microbial constituent colonizes the gastrointestinal tract—further modulating the microbiome and health of the host [30,31].

The findings of this study demonstrating that *Akkermansia* appears to be most prevalent among pediatric samples may be particularly relevant given that recent evidence suggesting that microbial diversity including *Akkermansia* is strongly associated with metabolic health in children, particularly among those who are overweight and obese [32,33]. In addition, these results combined with the results of the only other study to date evaluating microbial prevalence of *Akkermansia* among orthodontic patients, greatly increases the overall number of patients evaluated with and without orthodontic appliances [34]. Research has demonstrated that orthodontic therapy shifts oral microbial composition particularly among overweight and obese, adolescent patients [35]. These results may provide some of the first observations of these shifts among this specific patient population. In fact, recent studies from this group have found that body mass index (BMI) has been steadily increasing among the pediatric and adolescent patient population, with most recent average BMI ranging between 25.6 (overweight) and 31.3 (obese) [36,37].

Additional studies from this group have evaluated the role of orthodontic brackets in altering microbial populations, including *Selenomonas noxia*, *Scardovia wiggsiae*, *Streptococcus mutans* and *Porphyomonas gingivalis* [23,24,25,38,39]. The comparative analysis undertaken in this current study demonstrates that age-related changes in the oral prevalence of Gram-negative *Akkermansia* are similar to that of Gram-negative *Selenomonas* (higher levels among children than adults) but may exhibit opposite change with the presence of orthodontic appliances (*Akkermansia* decreased, *Selenomonas* increased), confirmed by other reports from this group reporting *Selenomonas* prevalence among orthodontic and non-orthodontic patients [26,27]. Furthermore, the finding that *Akkermansia* prevalence decreased among orthodontic patients while the periodontal pathogens *Fusobacterium* and *Porphyromonas* increased, may suggest that the mechanisms that drive these changes may be separate and distinct from changes in the periodontium and gingival crevices normally observed in orthodontic therapy [28,29].

Despite the significance of these findings, there are several limitations of this study which should also be considered. First, this is a retrospective study of clinical samples from an existing biorepository that may have some pre-existing differences in demographics due to the sample collection protocols of those initial study sample collection protocols [22,23,24]. In addition, most of the clinical patient population at this publicly funded university-based dental school are low-income and minority patients that may also have additional challenges and barriers to access and care that may have influenced the outcomes of this study—although no clinical measures such as periodontal pocket depth (PPD), plaque index (PI) or decayed-missing-filled teeth (DMFT) score were available among these retrospective samples for analysis [25,38,39]. Some evidence from this institution also suggests that oral microbial prevalence may be strongly shifting over time, which was not a primary outcome variable analyzed for in the current study and may have influenced these findings [40]. Due to the limited scale of this project and limited financial support for this preliminary study, other methodologies such as next generation sequencing were not employed—although this was unlikely to affect the outcome of this study. Finally, research has demonstrated that medically compromised patients may also suffer from changes in microbial load and may be an important patient population for future studies of this nature [41].

## 5. Conclusions

This study provides strong evidence that *Akkermansia* prevalence shifts with age, with younger patients more likely to harbor detectable levels in saliva that could potentially seed the gastrointestinal tract and influence gut microbial composition later in life. In addition, this study found that presence of orthodontics dramatically shifted *Akkermansia* prevalence among both pediatric and adult populations, which did not correlate with shifts of other known periodontal pathogens. This provides some of the first evidence that orthodontic therapy may be associated with changes in oral *Akkermansia* prevalence but may be related to the shifts in other oral microbial communities rather than the known changes in gingivitis and periodontitis typically associated with orthodontic treatment.

## Figures and Tables

**Table 1:** Demographic analysis of pediatric study samples.

Demographic	Pediatric Study Sample (*n* = 141)	Clinic Population	Statistical Analysis
*Sex*			
Pediatric—Female	(*n* = 72/141) 51.1%	52.8%	χ^2^ = 0.160, d.f. = 1
Pediatric—Male	(*n* = 69/141) 48.9%	47.2%	*p* = 0.6891
*Race/Ethnicity*			
Pediatric—White	(*n* = 42/141) 29.8%	24.7%	χ^2^ = 1.333, d.f. = 1
Pediatric—Minority	(*n* = 99/141) 70.2%	75.3%	*p* = 0.2482
Pediatric—Hispanic	(*n* = 74/141) 52.5%	52.1%	
*Age*			
Average (Median)	13.14 yrs. (12 yrs.)	10.44 yrs. (10 years)	Two-tailed *t*-test *p* = 0.021
Range	7–17 yrs.	0–17 yrs.	

**Table 2:** Demographic analysis of adult study samples.

Demographic	Adult Study Sample (*n* = 86)	Clinic Population	Statistical Analysis
*Sex*			
Adult—Female	(*n* = 42/86) 48.8%	49.1%	χ^2^ = 0.016, d.f. = 1
Adult—Male	(*n* = 44/86) 51.2%	50.9%	*p* = 0.9883
*Race/Ethnicity*			
Adult—White	(*n* = 32/86) 37.2%	34.6%	χ^2^ = 2.987, d.f. = 1
Adult—Minority	(*n* = 54/86) 62.8%	65.4%	*p* = 0.0839
Adult—Hispanic	(*n* = 47/86) 54.7%	58.6%	
*Age*			
Average (Median)	46.35 yrs. (42 yrs.)	42.31 yrs. (41 yrs.)	Two-tailed *t*-test *p* = 0.028
Range	18–73 yrs.	18–89 yrs.	

**Table 3:** Analysis of DNA from study samples.

Study Sample	DNA Concentration	DNA Purity (A260:A280 Ratio)
Pediatric samples *n* = 141	481.2 ng/uL +/−55.1 127.2–769.1 ng/uL	Average: 1.73 Range: 1.69–1.82
Adult samples *n* = 86	434.1 ng/uL +/−72.2 169.3–872.8 ng/uL	Average: 1.75 Range: 1.71–1.84

**Table 4:** Analysis of AM-positive and AM-negative pediatric samples.

Demographic	AM-Positive	AM-Negative	Statistical Analysis
Pediatric–Males	*n* = 21/69 or 30.4%	*n* = 48/69 or 69.6%	χ^2^ = 0.044, d.f. = 1
Pediatric—Females	*n* = 21/72 or 29.2%	*n* = 51/72 or 70.8%	*p* = 0.8347
Total	*n* = 42/141 or 29.8%	*n* = 99/141 or 70.2%	
Pediatric—Males Non-Orthodontic	*n* = 16/38 or 42.1%	*n* = 22/38 or 57.9%	χ^2^ = 758.853, d.f. = 1
Pediatric—Males Orthodontic	*n* = 5/31 or 16.1%	*n* = 26/31 or 83.9%	*p* = 0.0001
Total	*n* = 21/69 or 30.4%	*n* = 48/69 or 69.6%	
Pediatric—Females Non-Orthodontic	*n* = 17/40 or 42.5%	*n* = 23/40 or 57.5%	χ^2^ = 822.857, d.f. = 1
Pediatric—Females Orthodontic	*n* = 4/32 or 12.5%	*n* = 28/32 or 87.5%	*p* = 0.0001
Total	*n* = 21/72 or 29.2%	*n* = 51/72 or 70.8%	
Non-Orthodontic	*n* = 33/78 or 42.3%	*n* = 45/78 or 57.6%	χ^2^ = 639.734, d.f. = 1
Orthodontic	*n* = 9/63 or 14.3%	*n* = 54/63 or 85.7%	*p* = 0.0001
Total	*n* = 42/141 or 29.8%	*n* = 99/141 or 70.2%	

**Table 5:** Analysis of AM-positive and AM-negative adult samples.

Demographic	AM-Positive	AM-Negative	Statistical Analysis
Adult–Males	*n* = 8/44 or 18.2%	*n* = 36/44 or 81.8%	χ^2^ = 1.617, d.f. = 1
Adult—Females	*n* = 7/42 or 16.7%	*n* = 35/42 or 83.3%	*p* = 0.2035
Total	*n* = 15/86 or 17.4%	*n* = 71/86 or 82.6%	
Adult—Males Non-Orthodontic	*n* = 5/24 or 20.8%	*n* = 19/24 or 79.2%	χ^2^ = 26.384, d.f. = 1
Adult—Males Orthodontic	*n* = 3/20 or 15.0%	*n* = 17/20 or 85.0%	*p* = 0.0001
Total	*n* = 8/44 or 18.2%	*n* = 36/44 or 81.8%	
Adult—Females Non-Orthodontic	*n* = 5/23 or 21.7%	*n* = 18/23 or 78.3%	χ^2^ = 133.482, d.f. = 1
Pediatric—Females Orthodontic	*n* = 2/19 or 10.5%	*n* = 17/19 or 89.5%	*p* = 0.0001
Total	*n* = 7/42 or 16.7%	*n* = 35/42 or 83.3%	
Non-Orthodontic	*n* = 10/47 or 21.3%	*n* = 37/47 or 78.7%	χ^2^ = 64.731, d.f. = 1
Orthodontic	*n* = 5/39 or 12.8%	*n* = 34/39 or 87.2%	*p* = 0.0001
Total	*n* = 15/86 or 17.4%	*n* = 71/86 or 82.6%	

## Data Availability

The data presented in this study are available on request from the corresponding author. The data are not publicly available due to the study protocol data protection parameters requested by the IRB and OPRS for the initial study approval.

## References

[1] Karamzin A.M., Ropot A.V., Sergeyev O.V., Khalturina E.O. (2021). *Akkermansia muciniphila* and host interaction within the intestinal tract. Anaerobe.

[2] Geerlings S.Y., Kostopoulos I., de Vos W.M., Belzer C. (2018). *Akkermansia muciniphila* in the Human Gastrointestinal Tract: When, Where, and How?. Microorganisms.

[3] Aggarwal V., Sunder S., Verma S.R. (2022). Disease-associated dysbiosis and potential therapeutic role of *Akkermansia muciniphila*, a mucus degrading bacteria of gut microbiome. Folia. Microbiol..

[4] de Vos W.M., Tilg H., Van Hul M., Cani P.D. (2022). Gut microbiome and health: Mechanistic insights. Gut.

[5] Michels N., Zouiouich S., Vanderbauwhede B., Vanacker J., Indave Ruiz B.I., Huybrechts I. (2022). Human microbiome and metabolic health: An overview of systematic reviews. Obes. Rev..

[6] Vallianou N., Christodoulatos G.S., Karampela I., Tsilingiris D., Magkos F., Stratigou T., Kounatidis D., Dalamaga M. (2021). Understanding the Role of the Gut Microbiome and Microbial Metabolites in Non-Alcoholic Fatty Liver Disease: Current Evidence and Perspectives. Biomolecules.

[7] Xu Y., Wang N., Tan H.Y., Li S., Zhang C., Feng Y. (2020). Function of *Akkermansia muciniphila* in Obesity: Interactions With Lipid Metabolism, Immune Response and Gut Systems. Front. Microbiol..

[8] Macchione I.G., Lopetuso L.R., Ianiro G., Napoli M., Gibiino G., Rizzatti G., Petito V., Gasbarrini A., Scaldaferri F. (2019). *Akkermansia muciniphila*: Key player in metabolic and gastrointestinal disorders. Eur. Rev. Med. Pharmacol. Sci..

[9] Cheng H.L., Yen G.C., Huang S.C., Chen S.C., Hsu C.L. (2022). The next generation beneficial actions of novel probiotics as potential therapeutic targets and prediction tool for metabolic diseases. J. Food Drug Anal..

[10] Dudík B., Kiňová Sepová H., Greifová G., Bilka F., Bílková A. (2022). Next generation probiotics: An overview of the most promising candidates. Epidemiol. Mikrobiol. Imunol..

[11] Shen H., Zhao Z., Zhao Z., Chen Y., Zhang L. (2022). Native and Engineered Probiotics: Promising Agents against Related Systemic and Intestinal Diseases. Int. J. Mol. Sci..

[12] Minty M., Canceil T., Serino M., Burcelin R., Tercé F., Blasco-Baque V. (2019). Oral microbiota-induced periodontitis: A new risk factor of metabolic diseases. Rev. Endocr. Metab. Disord..

[13] Graves D.T., Corrêa J.D., Silva T.A. (2019). The Oral Microbiota Is Modified by Systemic Diseases. J. Dent. Res..

[14] Huck O., Mulhall H., Rubin G., Kizelnik Z., Iyer R., Perpich J.D., Haque N., Cani P.D., de Vos W.M., Amar S. (2020). *Akkermansia muciniphila* reduces *Porphyromonas gingivalis*-induced inflammation and periodontal bone destruction. J. Clin. Periodontol..

[15] Tavares C.O., Rost F.L., Silva R.B.M., Dagnino A.P., Adami B., Schirmer H., de Figueiredo J.A.P., Souto A.A., Maito F.D.M., Campos M.M. (2019). Cross Talk between Apical Periodontitis and Metabolic Disorders: Experimental Evidence on the Role of Intestinal Adipokines and *Akkermansia muciniphila*. J. Endod..

[16] Mulhall H., DiChiara J.M., Deragon M., Iyer R., Huck O., Amar S. (2020). *Akkermansia muciniphila* and Its Pili-Like Protein Amuc_1100 Modulate Macrophage Polarization in Experimental Periodontitis. Infect. Immun..

[17] Mulhall H., DiChiara J.M., Huck O., Amar S. (2022). Pasteurized *Akkermansia muciniphila* reduces periodontal and systemic inflammation induced by *Porphyromonas gingivalis* in lean and obese mice. J. Clin. Periodontol..

[18] Tarallo S., Ferrero G., De Filippis F., Francavilla A., Pasolli E., Panero V., Cordero F., Segata N., Grioni S., Pensa R.G. (2022). Stool microRNA profiles reflect different dietary and gut microbiome patterns in healthy individuals. Gut.

[19] Becken B., Davey L., Middleton D.R., Mueller K.D., Sharma A., Holmes Z.C., Dallow E., Remick B., Barton G.M., David L.A. (2021). Genotypic and Phenotypic Diversity among Human Isolates of *Akkermansia muciniphila*. mBio.

[20] Yan J., Sheng L., Li H. (2021). *Akkermansia muciniphila*: Is it the Holy Grail for ameliorating metabolic diseases?. Gut Microbes..

[21] Singh H., Torralba M.G., Moncera K.J., DiLello L., Petrini J., Nelson K.E., Pieper R. (2019). Gastro-intestinal and oral microbiome signatures associated with healthy aging. Geroscience.

[22] Emett J., David R., McDaniel J., McDaniel S., Kingsley K. (2020). Comparison of DNA Extracted from Pediatric Saliva, Gingival Crevicular Fluid and Site-Specific Biofilm Samples. Methods Protoc..

[23] McDaniel J., McDaniel S., Samiano B.J., Marrujo M., Kingsley K., Howard K.M. (2021). Microbial Screening Reveals Oral Site-Specific Locations of the Periodontal Pathogen *Selenomonas noxia*. Curr. Issues Mol. Biol..

[24] McDaniel S., McDaniel J., Howard K.M., Kingsley K. (2021). Molecular Screening and Analysis Reveal Novel Oral Site-Specific Locations for the Cariogenic Pathogen *Scardovia wiggsiae*. Dent. J..

[25] Davis J.E., Freel N., Findley A., Tomlin K., Howard K.M., Seran C.C., Cruz P., Kingsley K. (2012). A molecular survey of S. mutans and P. gingivalis oral microbial burden in human saliva using relative endpoint polymerase chain reaction (RE-PCR) within the population of a Nevada dental school revealed disparities among minorities. BMC Oral Health.

[26] Dhillon N., Kingsley K., Howard K.M. (2019). Prevalence of *Selenomonas noxia* among Pediatric and Adult Orthodontic Patients. Int. J. Res. Rep. Dent..

[27] Kim N., Trumbo M., Perkins P., Foote K., Samiano B.J., Marrujo M., Kingsley K., Howard K.M. (2021). Comparing the Incidence and Prevalence of Oral Microbial Pathogens *Selenomonas noxia* and Streptococcus mitis within the UNLV-SDM Clinical Patient Population. Int. J. Res. Rep. Dent..

[28] Kingsley J., Kingsley K. (2019). *Aggregatibacter actinomycetemcomitans* and *Fusobacterium nucleatum* prevalence correlates with salivary microbial burden in Orthodontic patients. Int. J. Dent. Res. Rev..

[29] Klingler J. (2019). Prevalence of *Aggregatibacter actinomycetemcomitans* and *Fusobacterium nucleatum* among Clinical Orthodontic and Non-Orthodontic Saliva Samples. Doctoral Dissertation.

[30] de Vos W.M. (2017). Microbe Profile: *Akkermansia muciniphila*: A conserved intestinal symbiont that acts as the gatekeeper of our mucosa. Microbiology.

[31] Luo Y., Lan C., Li H., Ouyang Q., Kong F., Wu A., Ren Z., Tian G., Cai J., Yu B. (2022). Rational consideration of *Akkermansia muciniphila* targeting intestinal health: Advantages and challenges. npj Biofilms Microbiomes.

[32] Alcazar M., Escribano J., Ferré N., Closa-Monasterolo R., Selma-Royo M., Feliu A., Castillejo G., Luque V., Feliu-Rovira A., Muñoz-Hernando J. (2022). Gut microbiota is associated with metabolic health in children with obesity. Clin. Nutr..

[33] Sugino K.Y., Ma T., Paneth N., Comstock S.S. (2021). Effect of Environmental Exposures on the Gut Microbiota from Early Infancy to Two Years of Age. Microorganisms.

[34] Wirth R., Maróti G., Lipták L., Mester M., Al Ayoubi A., Pap B., Madléna M., Minárovits J., Kovács K.L. (2022). Microbiomes in supragingival biofilms and saliva of adolescents with gingivitis and gingival health. Oral Dis..

[35] Sharara S.H., Cleaver L.M., Saloom H., Carpenter G.H., Cobourne M.T. (2022). Salivary bacterial community profile in normal-weight and obese adolescent patients prior to orthodontic treatment with fixed appliances. Orthod. Craniofac. Res..

[36] Su T., Jackson M., Sacry K., Kingsley K. (2022). Longitudinal Analysis of Body Mass Index (BMI) Trends among a Pediatric Dental Patient Population. EC Paediatr..

[37] Frayna C., Devantier C., Harris B., Kingsley K., Polanski J.M. (2021). Education Regarding and Adherence to Recommended Nutrition Guidelines among Dental Students. Dent. J..

[38] Milne W., Rezaei G., Whiteley A., Kingsley K. (2018). Cariogenic pathogen *Scardovia wiggsiae* screening among pediatric orthodontic patients: A pilot Study. Curr. Res. Dent..

[39] Reyes N., Pollock A., Whiteley A., Kingsley K., Howard K.M. (2017). Prevalence of *Scardovia wiggsiae* among a pediatric Orthodontic patient population. EC Dent. Sci..

[40] Kornhaber M.S., Florence T., Davis T., Kingsley K. (2022). Assessment of Oral Human Papillomavirus Prevalence in Pediatric and Adult Patients within a Multi-Ethnic Clinic Population. Dent. J..

[41] Pawlaczyk-Kamieńska T., Borysewicz-Lewicka M., Batura-Gabryel H. (2019). Salivary Biomarkers and Oral Microbial Load in Relation to the Dental Status of Adults with Cystic Fibrosis. Microorganisms.

